# Effect of onion on blood lipid profile: A meta‐analysis of randomized controlled trials

**DOI:** 10.1002/fsn3.2309

**Published:** 2021-05-13

**Authors:** Wang Huang, Gang Tang, Linyu Zhang, Jie Tao, Zhengqiang Wei

**Affiliations:** ^1^ Department of Gastrointestinal Surgery The First Affiliated Hospital of Chongqing Medical University Chongqing China; ^2^ Department of Clinical Medicine Chongqing Medical University Chongqing China

**Keywords:** cholesterol, lipid profiles, meta‐analysis, onion, triglycerides

## Abstract

**Background:**

Studies indicate that onion supplementation may be effective in the treatment of dyslipidemia; however, the results remain controversial. This meta‐analysis was conducted to evaluate potential benefits of onion on lipid profile.

**Methods:**

Up to 12 October 2020, PubMed, Cochrane Library, Web of Science, and Scopus were searched for randomized controlled trials evaluating the effects of onion on lipid profile. Mean differences (MD) and 95% confidence intervals (CI) were calculated. Meta‐analysis was conducted using the fixed‐effects model.

**Results:**

Ten trials with 446 participants in total were included in the meta‐analysis. The pooled findings of 10 studies suggested that onion supplementation significantly improved high‐density lipoprotein cholesterol (HDL) (MD: 2.29 mg/dl; 95% CI: 0.87, 3.72; *I* ^2^ = 0%) and low‐density lipoprotein cholesterol (LDL) (MD: −6.64 mg/dl; 95% CI: −10.91, −2.36; *I* ^2^ = 32%),while onion supplementation did not significantly lower triglycerides (TG) (MD: −6.55 mg/dl; 95% CI: −15.64, 2.53; *I* ^2^ = 45%). Analysis of nine trials showed a significant reduction in total cholesterol (TC) (MD: −5.39 mg/dl; 95% CI: −10.68, −0.09; *I* ^2^ = 49%) in patients with onion supplementation compared to the control group.

**Conclusion:**

In summary, supplementation of onion was beneficial to control dyslipidemia, including improving levels of HDL, LDL, and TC, but could not reduce TG level. The therapeutic benefits of onion for dyslipidemia need to be treated with caution considering that some of the results are not robust.

## Graphical abstract

Cardiovascular diseases are, currently, the leading cause of death globally, accounting for about one third of all deaths. Controlling blood lipids can effectively reduce the risk of cardiovascular diseases. Onion supplementation was beneficial for blood lipids, including improving plasma levels of HDL, LDL, and TC.

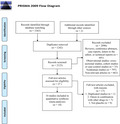

## 1 INTRODUCTION

Cardiovascular diseases are currently the leading cause of death globally, accounting for about one‐third of all deaths (Hadjiphilippou & Ray, 2019; Yusuf et al., 2020). Dyslipidemia is a major risk factor for the development and progression of cardiovascular diseases (Kopin & Lowenstein, 2017; Pan et al., 2016). Dyslipidemia is an incurable but controllable condition characterized by elevated plasma triglycerides (TG), total cholesterol (TC), or low‐density lipoprotein cholesterol (LDL) concentrations and decreased plasma high‐density lipoprotein cholesterol (HDL) concentrations (Hedayatnia et al., 2020; Heshmat‐Ghahdarijani et al., 2020). Previous studies have shown that controlling blood lipids can effectively reduce the risk of cardiovascular diseases (Ida et al., 2019). Therefore, the treatment of dyslipidemia is one of the keys to prevent cardiovascular diseases (Yandrapalli et al., 2019). Statins, the main lipid‐lowering agents, are effective in improving blood lipids, but they are associated with many side effects that affect patient compliance (Karr, 2017). Furthermore, the reduction of drug efficacy is also a problem that cannot be ignored when statins are used for a long‐term (Shekarchizadeh‐Esfahani et al., 2020). Therefore, it is urgent to find a safe and effective supplementary treatment.

Alternatives based on natural products are of great interest because of their safety and potential effectiveness (Georgia‐Eirini et al., 2019; Yarla et al., 2016). Onion (*Allium Cepa* L.) is a perennial plant, belonging to Family Amaryllidaceae, one of the most widely consumed vegetables in the world (Khajah et al., 2019; Li et al., 2020). Onions are rich in sulfur compounds and Flavonoids (Chiu et al., 2016), and have traditionally been used to treat asthma, coughs, high blood pressure, ulcer wounds, and other ailments (Khajah et al., 2019). In recent years, onion has been found to have many kinds of biological activities, including anti‐inflammatory, anti‐oxidant, anticancer, anti‐diabetes, Immunoprotective, wound healing, anti‐scar and, anti‐obesity, and widely used in medicine (Khajah et al., 2019; Teshika et al., 2019). Recently, several studies have examined the effects of onion on blood lipids, but the results are contradictory. Lee et al. (2011) found that supplementation with 228 mg onion skin extract for 10 weeks significantly improved TC, LDL, and HDL levels in healthy subjects, but in Kim et al.'s study (2015), there was no significant difference in lipid profile after 12 weeks of administration of onion peel extract of 100 mg/day compared with placebo.

Therefore, it is necessary to summarize the evidence of the effects of onion on blood lipids. Unfortunately, there has been no systematic review or meta‐analysis in this area. The purpose of this study was to comprehensively review randomized controlled trials to evaluate the effects of onion on lipid profile.

## 2 METHODS AND MATERIALS

### 2.1 Search strategy

Two authors (Tang and Tao) independently searched the literature through PubMed, Cochrane Library, Web of Science, and Scopus from inception to 12 October 2020. Search strategy (Table 1): (*Allium Cepa* OR onion) AND (lipid OR lipids OR hyperlipidemic OR hyperlipidemia OR dyslipidemic OR dyslipidemia OR cholesterol OR hypercholesterolemic OR hypercholesterolemia OR triglycerides OR Triacylglycerol OR hypotriglyceridemic OR hypertriglyceridemia OR lipoprotein OR lipoproteins OR TG OR TC OR LDL OR HDL). Furthermore, references of the included studies were searched to reduce possible omissions.

**TABLE 1:** Electronic search strategy

Database	Search term (establish to 12 October 2020)	Number
PubMed (All fields)	#1: Allium Cepa OR onion	#1: 7,887
	#2: lipid OR lipids OR hyperlipidemic OR hyperlipidemia OR dyslipidemic OR dyslipidemia OR cholesterol OR hypercholesterolemic OR hypercholesterolemia OR triglycerides OR Triacylglycerol OR hypotriglyceridemic OR hypertriglyceridemia OR lipoprotein OR lipoproteins OR TG OR TC OR LDL OR HDL	#2: 1,685,442
	#3: #1 AND #2	#3: 783
Scopus (TITLE‐ABS‐KEY)	#1: Allium Cepa OR onion	#1: 23,917
	#2: lipid OR lipids OR hyperlipidemic OR hyperlipidemia OR dyslipidemic OR dyslipidemia OR cholesterol OR hypercholesterolemic OR hypercholesterolemia OR triglycerides OR Triacylglycerol OR hypotriglyceridemic OR hypertriglyceridemia OR lipoprotein OR lipoproteins OR TG OR TC OR LDL OR HDL	#2: 1,668,539
	#3: #1 AND #2	#3: 1,010
Cochrane Library Trials (Title Abstract keyword)	#1: Allium Cepa OR onion	#1: 240
	#2: lipid OR lipids OR hyperlipidemic OR hyperlipidemia OR dyslipidemic OR dyslipidemia OR cholesterol OR hypercholesterolemic OR hypercholesterolemia OR triglycerides OR Triacylglycerol OR hypotriglyceridemic OR hypertriglyceridemia OR lipoprotein OR lipoproteins OR TG OR TC OR LDL OR HDL	#2: 78,607
	#3: #1 AND #2	#3: 41
Web of Science (Topic)	#1: Allium Cepa OR onion	#1: 21,518
	#2: lipid OR lipids OR hyperlipidemic OR hyperlipidemia OR dyslipidemic OR dyslipidemia OR cholesterol OR hypercholesterolemic OR hypercholesterolemia OR triglycerides OR Triacylglycerol OR hypotriglyceridemic OR hypertriglyceridemia OR lipoprotein OR lipoproteins OR TG OR TC OR LDL OR HDL	#2: 1,971,491
	#3: #1 AND #2	#3: 1,529

### 2.2 Study selection

Trials meeting the following criteria were included: (a) Participants with dyslipidemia or without dyslipidemia; (b) Intervention were onion or products of onion; (c) The control group was placebo; (d) Outcomes assessed at least one of LDL, HDL, TG, or TC; (e) The study was designed as a randomized controlled trial. Exclusion criteria are as follows: (a) non‐human studies; (b) reviews, conference abstracts, case reports, letters to the editor, or technical reports; (c) reported on observational studies; (d) duplicate studies; (e) onion in combination with other drugs.

### 2.3 Data extraction

Characteristics of the included study were extracted, including first author, publication year, location, age, sample size, baseline body mass index (BMI), state of health, type of study, intervention, duration, outcomes. In addition, the mean changes of TG, TC, HDL, and LDL from baseline to the end of the study and their standard deviations (SDs) were also extracted. When data in the literature was not available, we contacted the corresponding author and attempt to obtain the data.

### 2.4 Quality assessment

The whole process of literature search, screening, data extraction, and quality assessment was conducted by two authors (Tang and Tao) independently, and all inconsistencies were discussed with the third author (Zhang) and resolved. Cochrane Collaboration's tool was used to conduct quality assessments, including the following seven domains: (a) random sequence generation; (b) allocation concealment; (c) blinding of participants and personnel; (d) blinding of outcome assessment; (e) incomplete outcome data; (f) selective reporting; (g) other sources of bias. Each domain was classified as high bias risk, low bias risk, and unclear bias risk. Based on the domains mentioned, the overall quality of each study was assessed as good (more than two domains were assessed as low risk), fair (two domains were assessed as low risk), or weak (less than two domains were assessed as low risk).

### 2.5 Statistical analysis

All concentration units were converted to mg/dl before the total effect is calculated. Effect size was defined as mean difference (MD) and 95% confidence interval (CI) (Higgins & Green, 2011. Available from www.cochrane‐handbook.org). When no net change in the outcomes (TG, TC, LDL, and HDL) was provided, the difference between baseline and end is calculated as the effect size. The following formula was used to calculate standard deviations (SDs) of the mean changes: *SD* = square root [(SD_pretreatment_)^2^ + (SD_posttreatment_)^2^ ‐ 2r × SD_pretreatment_ × SD_posttreatment_], correlation coefficient (*r*) = .5. When standard error (*SE*) was reported, the following formula is used to calculate *SD*: *SD* = *SE* × √*n* (*n* = number of subjects). *I* ^2^ statistics were used to assess the size of heterogeneity. When *I* ^2^ > 50 was significant heterogeneity, and the random‐effect model was selected, whereas the fixed‐effect model was selected (Higgins et al., 2003). We separately excluded each study to explore the robustness of our results. Begg's rank correlation and Egger's weighted regression were used to examine potential publication bias. Subgroup analysis was performed based on the type of participants (dyslipidemia or without dyslipidemia) and period of treatment (>10 weeks or ≤10 weeks). All statistical analyses were performed using Review Manager 5.3 (The Nordic Cochrane Centre, The Cochrane Collaboration 2014; Copenhagen, Denmark) and Stata 12.0 (Stata Corp.). Moreover, statistically significant is defined as *p* < .05.

## 3 RESULTS

### 3.1 Identification and selection of studies

**FIGURE. 1:**
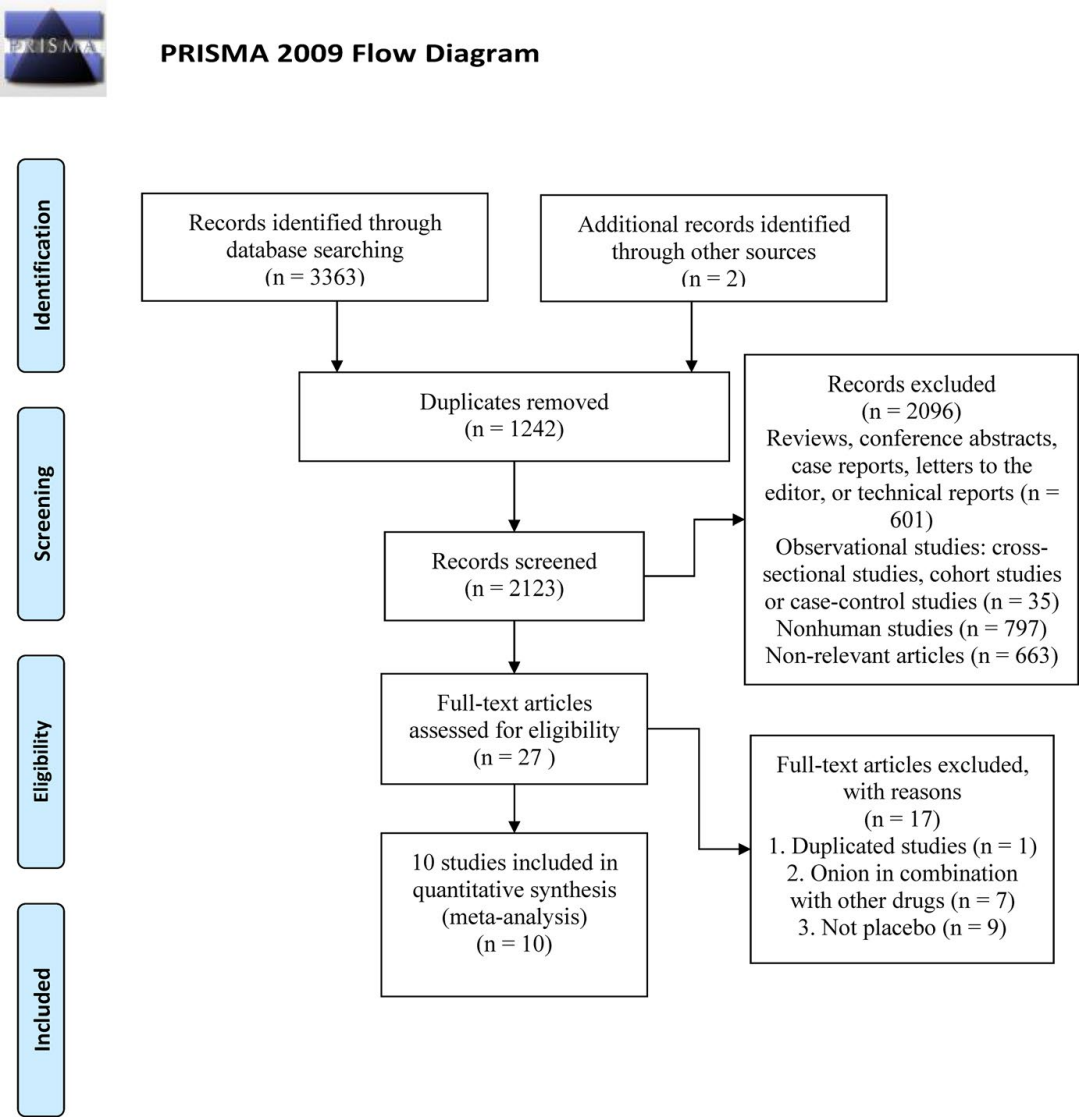
PRISMA flow diagram of the literature retrieval process

Of the 3,365 related literatures, 1,242 duplicates were excluded. Next, 2,096 literatures that did not meet the inclusion criteria were excluded by reviewing the title and abstract. Then, we evaluated the remaining 27 articles, and finally, 10 articles were included (Figure 1).

### 3.2 Study characteristics

A total of 446 participants in the 10 included studies were randomly assigned to either the placebo group or the onion group. The number of participants in each study ranged from 12 to 92. Two studies (Kim et al., 2013; Lee et al., 2011) involved healthy adult, three studies involved hyperlipidemia subjects (Kyung Huil et al., 2007; Lee et al., 2010; Lu et al., 2015), and five studies involved Overweight and/or obesity subjects (Bruell et al., 2016; Choi et al., 2015; Jeong et al., 2019; Kim & Yim, 2015; Park et al., 2016). All the studies were published between 2007 and 2019. Eight trails (Choi et al., 2015; Jeong et al., 2019; Kim et al., 2013; Kim & Yim, 2015; Kyung Huil et al., 2007; Lee et al., 2010; Lee et al., 2011; Park et al., 2016) were conducted in Korea, one (Lu et al., 2015) in China and one (Bruell et al., 2016) in Germany. The duration of intervention in the included studies ranged from 2 to 12 weeks. Seven trials (Choi et al., 2015; Jeong et al., 2019; Kim & Yim, 2015; Kyung Huil et al., 2007; Lee et al., 2011; Lu et al., 2015; Park et al., 2016) were designed in parallel and three (Bruell et al., 2016; Kim et al., 2013; Lee et al., 2010) in a cross‐over design. Characteristics of the included study are summarized in Table 2.

**TABLE 2:** Characteristics of all eligible studies

First author, publication year	Location	Age	Sample size	BMI	Subjects	Type of study	Intervention	Duration	outcomes
Kyung Hui (2007)	Korea	47.1	43	25.6	Patients with hypercholesterolemia	Randomized placebo‐controlled parallel trial	200 ml/day onion extract	8 weeks	TG, TC, HDL, LDL
Lee (2010)	Korea	45.9	27	24.6	borderline hypercholesterolemic subjects	Randomized single blind placebo‐controlled crossover trial	150 ml/day onion extract	10 weeks	TG, TC, HDL, LDL
Lee (2011)	Korea	44.4	92	24.9	Healthy male smokers	Randomized, Double blinded placebo‐controlled parallel trial	1,000 mg/day OPE	10 weeks	TG, TC, HDL, LDL
Kim (2013)	Korea	*N*	12	20.2	healthy young women	Randomized double‐blind placebo‐controlled crossover trial	1,000 mg/day OPE	2 weeks	TG, HDL, LDL
Kim (2015)	Korea	45	37	26.3	Obese women	Randomized double‐blind placebo controlled parallel trial	100 mg/day OPE	12 weeks	TG, TC, HDL, LDL
Choi (2015)	Korea	43.1	62	26.2	Overweight and obese subjects	Randomized double‐blind placebo‐controlled parallel trial	1,000 mg/day OPE	12 weeks	TG, TC, HDL, LDL
Lu (2015)	China	*N*	23	25.2	Mild hypercholesterolemic adults	Randomized placebo‐controlled parallel trial	100 ml/day onion juice	8 weeks	TG, TC, HDL, LDL
Brüll (2016)	Germany	48.1	22	30.9	Overweight‐to‐obese adults with hypertension	Randomized double‐blinded placebo‐controlled crossover trial	396 mg/day OPE	6 weeks	TG, TC, HDL, LDL
Park (2016)	Korea	43.3	72	26.6	Overweight and obese subjects	Randomized double‐blind, placebo‐controlled parallel trial	340 mg/day OPE	12 weeks	TG, TC, HDL, LDL
Jeong (2019)	Korea	42.0	56	27.2	Overweight Subjects	Randomized double‐blind placebo‐controlled parallel trial	900 mg/day steamed onion	12 weeks	TG, TC, HDL, LDL

Abbreviations: HDL, high‐density lipoprotein cholesterol; LDL, low‐density lipoprotein cholesterol; N, not available; OPE, onion peel extract; TG, Triglyceride; TC, total cholesterol.

### 3.3 Quality assessment

**FIGURE. 2:**
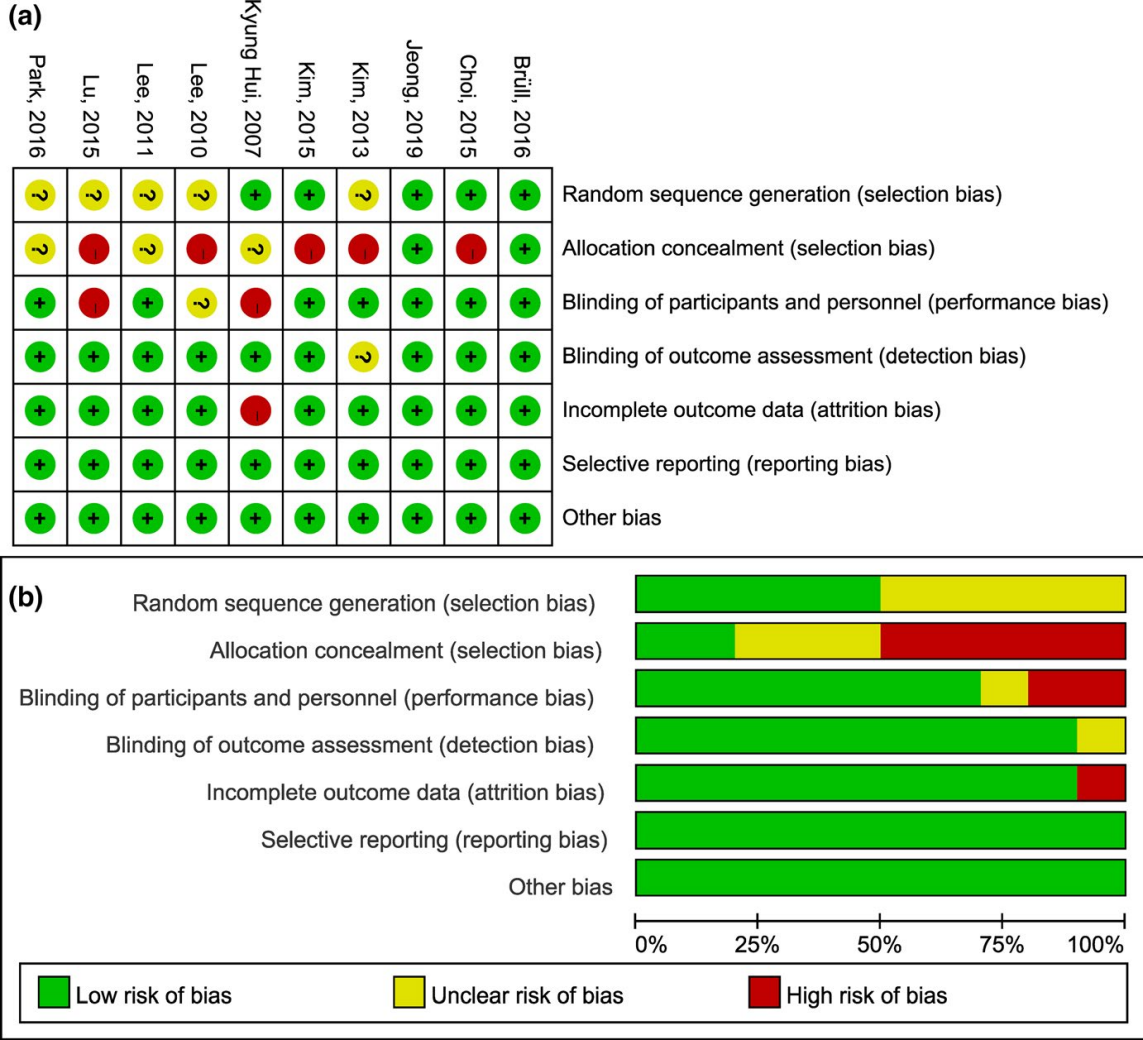
Risk of bias for each included study. (a) risk of bias summary; (b) risk of bias graph

All of the included studies mentioned randomization, but only five (Bruell et al., 2016; Choi et al., 2015; Jeong et al., 2019; Kim & Yim, 2015; Kyung Huil et al., 2007) provided specific methods for randomization. Two studies (Bruell et al., 2016; Jeong et al., 2019) adequately concealed the randomization scheme. A double‐blind design was used in seven studies (Bruell et al., 2016; Choi et al., 2015; Jeong et al., 2019; Kim et al., 2013; Kim & Yim, 2015; Kyung Huil et al., 2007; Lee et al., 2010; Lee et al., 2011; Lu et al., 2015; Park et al., 2016), blinding of outcome assessment in nine studies (Bruell et al., 2016; Choi et al., 2015; Jeong et al., 2019; Kim & Yim, 2015; Kyung Huil et al., 2007; Lee et al., 2010; Lee et al., 2011; Lu et al., 2015; Park et al., 2016) were considered as low risk of bias. Incomplete outcome data in nine studies (Bruell et al., 2016; Choi et al., 2015; Jeong et al., 2019; Kim et al., 2013; Kim & Yim, 2015; Lee et al., 2010; Lee et al., 2011; Lu et al., 2015; Park et al., 2016) were judged as low risk of bias. Selective reporting and other biases were assessed as low risk of bias in all studies. The risk of bias is shown in Figure 2.

### 3.4 Meta‐analysis

**FIGURE. 3:**
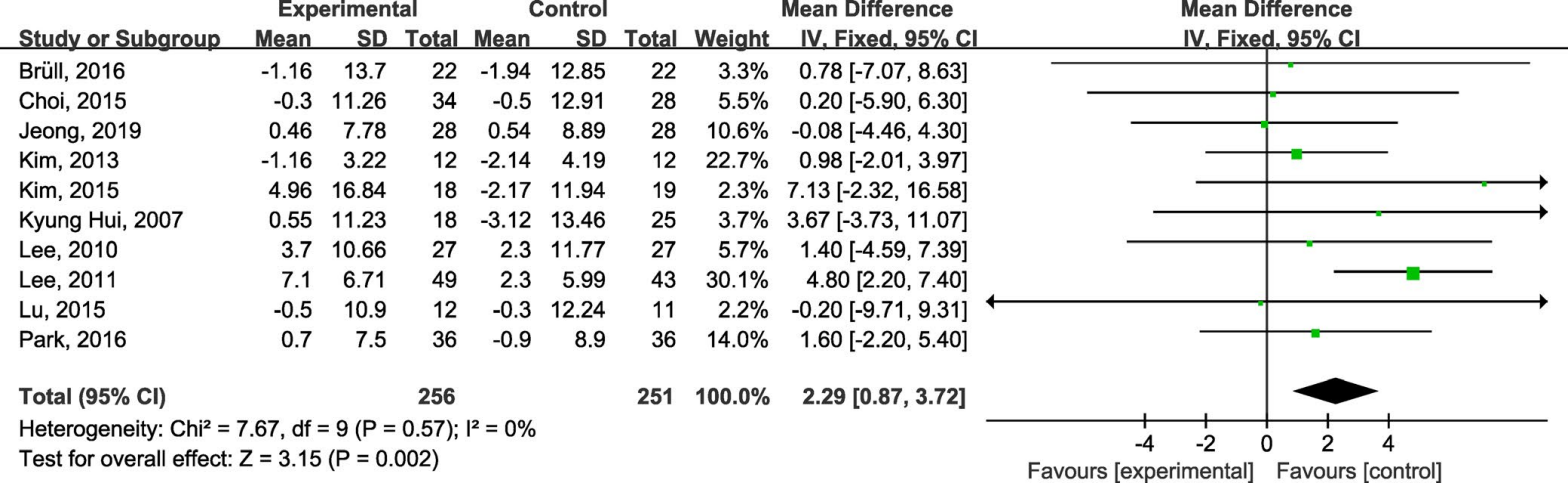
Forest plots of the effect of onion on plasma high‐density lipoprotein cholesterol concentrations

**FIGURE. 4:**
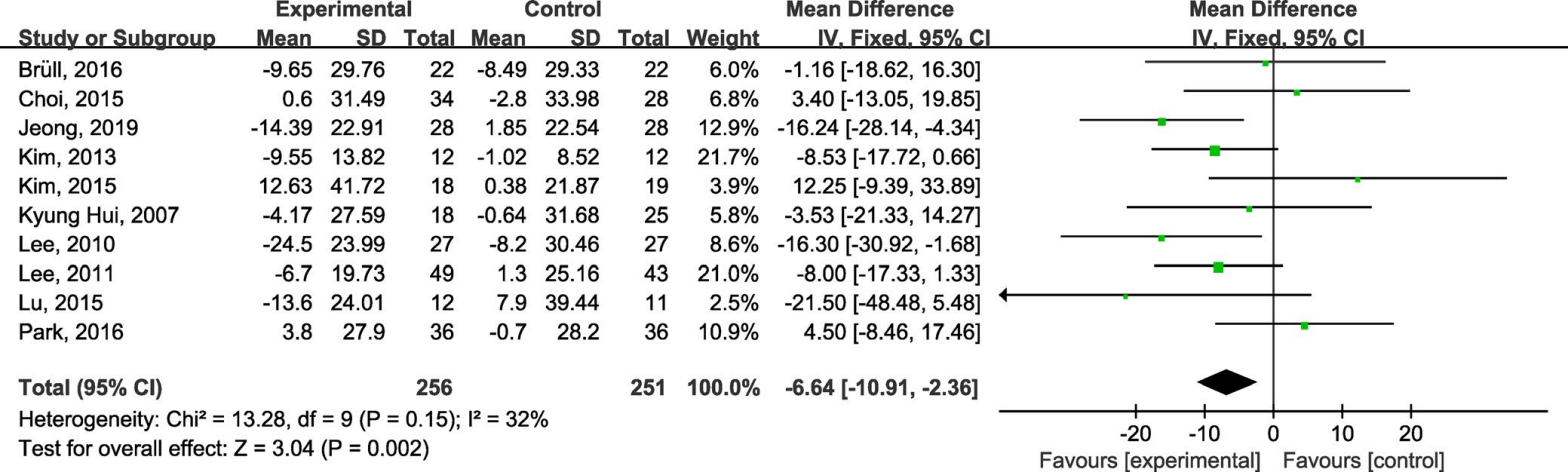
Forest plots of the effect of onion on plasma low‐density lipoprotein cholesterol concentrations

**FIGURE. 5:**
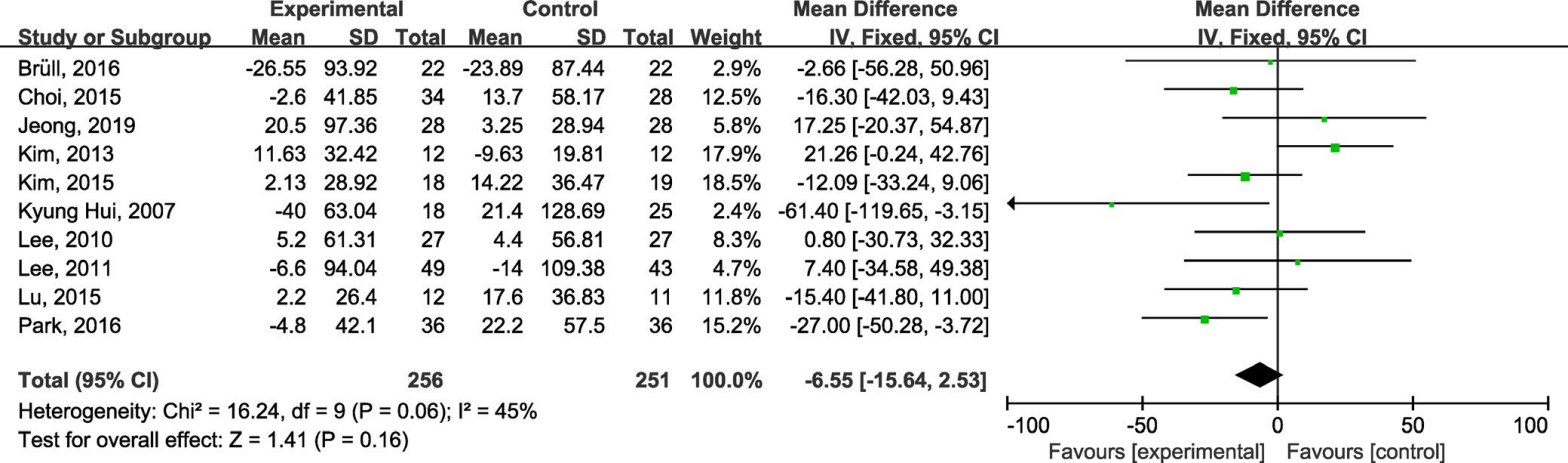
Forest plots of the effect of onion on plasma triglycerides concentrations

**FIGURE. 6:**
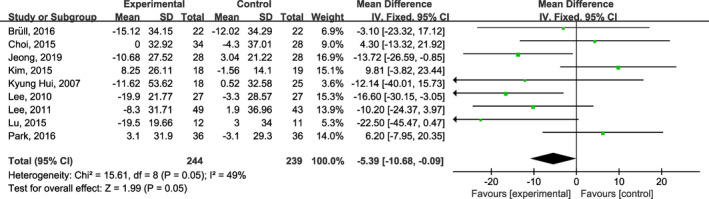
Forest plots of the effect of onion on plasma total cholesterol concentrations

Ten studies (Bruell et al., 2016; Choi et al., 2015; Jeong et al., 2019; Kim et al., 2013; Kim & Yim, 2015; Kyung Huil et al., 2007; Lee et al., 2010; Lee et al., 2011; Lu et al., 2015; Park et al., 2016) reported the effect of onion supplementation on lipid profiles. Pooled evaluation of the included trials (Bruell et al., 2016; Choi et al., 2015; Jeong et al., 2019; Kim et al., 2013; Kim & Yim, 2015; Kyung Huil et al., 2007; Lee et al., 2010; Lee et al., 2011; Lu et al., 2015; Park et al., 2016) indicated that onion supplementation significantly improved HDL (MD: 2.29 mg/dl; 95% CI: 0.87, 3.72; *I* ^2^ = 0%; Figure 3), LDL (MD: −6.64 mg/dl; 95% CI: −10.91, −2.36; *I* ^2^ = 32%; Figure 4), but no significant lowering in TG (MD: −6.55 mg/dl; 95% CI: −15.64, 2.53; *I* ^2^ = 45%; Figure 5). Moreover, and meta‐analysis of nine studies (Bruell et al., 2016; Choi et al., 2015; Jeong et al., 2019; Kim & Yim, 2015; Kyung Huil et al., 2007; Lee et al., 2010; Lee et al., 2011; Lu et al., 2015; Park et al., 2016) showed a significant effect on lowering TC (MD: −5.39 mg/dl; 95% CI: −10.68, −0.09; *I* ^2^ = 49%; Figure 6), as compared to the placebo group.

### 3.5 Subgroup analysis

Notably, the subgroup analysis showed that in the subgroup of subjects with dyslipidemia, onion intake appeared to show a greater benefit in lowering TC (MD: −17.23 mg/dl; 95% CI: −27.99, −6.47; *I* ^2^ = 8%) and LDL (MD: −12.70 mg/dl; 95% CI: −23.12, −2.27; *I* ^2^ = 0%). However, onion supplementation did not improve TG in either subjects with (MD: −14.4 mg/dl; 95% CI: −33.52, 4.72; *I* ^2^ = 41%) or without dyslipidemia (MD: −4.27 mg/dl; 95% CI: −14.59, 6.06; *I* ^2^ = 50%). In addition, we observed more increases in HDL concentrations (MD: 2.81 mg/dl; 95% CI: 1.08, 5.54; *I* ^2^ = 17%) in the subgroup with onion intake longer than 10 weeks. The results of subgroup analysis are summarized in Table 3.

**TABLE 3:** Summary of results from all subgroup analyses

‐	Subgrouped by	The number of studies	Effect size	95% CI	*I* ^2^ (%)	*p* for between subgroup heterogeneity
HDL	Type of participants	–	–	–	–	.81
	dyslipidemia	3	1.82	−2.37, 6.00	0	–
	without dyslipidemia	7	2.35	0.84, 3.87	16	–
	period of treatment	–	–	–	–	.30
	>10 weeks	6	2.81	1.08, 4.54	17	–
	≤10 weeks	4	1.19	−1.33, 3.71	0	–
LDL	Type of participants	–	–	–	–	.21
	dyslipidemia	3	−12.70	−23.12, −2.27	0	–
	without dyslipidemia	7	−5.41	−10.10, −0.72	40	–
	period of treatment	–	–	–	–	.79
	>10 weeks	6	−6.20	−11.55, −0.86	56	–
	≤10 weeks	4	−7.40	−14.54, −0.27	0	–
TG	Type of participants	–	–	–	–	.36
	dyslipidemia	3	−14.40	−33.52, 4.72	41	–
	without dyslipidemia	7	−4.27	−14.59, 6.06	50	–
	period of treatment	–	–	–	–	.22
	>10 weeks	6	−10.71	−21.98, 0.56	8	–
	≤10 weeks	4	−1.15	−14.20, 16.51	68	–
TC	Type of participants	–	–	–	–	.01
	dyslipidemia	3	−17.23	−27.99, −6.47	8	
	without dyslipidemia	6	−1.61	−7.69, 4.47	45	–
	period of treatment	–	–	–	–	.31
	>10 weeks	6	−4.20	−9.97, 1.56	62	–
	≤10 weeks	3	−11.70	−25.03, 1.63	0	–

Abbreviations: HDL, high‐density lipoprotein cholesterol; LDL, low‐density lipoprotein cholesterol; TC, total cholesterol; TG, Triglyceride.

### 3.6 Sensitivity analysis

Found of sensitivity analysis suggested that the pooled effect size of LDL was not affected by any of the studies. However, the effect of onion on level of TC was sensitive to the trials by Lu et al. (2015) (MD: −4.43; 95% CI: −9.87, 1.01), Lee et al. (2011) (MD: −4.61; 95% CI: −10.31, 1.10), Lee et al. (2010) (MD: −3.37; 95% CI: −9.12, 2.38), Kyung Hui et al. (2007) (MD: −5.13; 95% CI: −10.52, 0.26) and Jeong et al. (2019) (MD: −3.69; 95% CI: −9.50, 2.12), and Kim et al.’s study (2013) markedly affected the total effect size of TG (MD: −12.60; 95% CI: −22.63, −2.58). Sensitivity analysis indicated that the overall effect size of HDL was changed by excluding Lee et al.'s (2011) study (MD: 1.21; 95% CI: −0.49, 2.92).

### 3.7 Publication bias

There was no evidence of publication bias for TG (Begg's = 1.000, Egger's = 0.749), TC (Begg's = 0.917, Egger's = 0.703), LDL (Begg's = 0.592, Egger's = 0.481), and HDL (Begg's = 0.271, Egger's = 0.509).

## 4 DISCUSSION

To our knowledge, this is the first meta‐analysis to assess the impact of onion on blood lipids. Our results showed that onion could not reduce plasma TG levels, but onion administration did improve plasma HDL, TC, and LDL levels. In particular, for subjects with dyslipidemia, onion intake appeared to show a greater benefit in reducing TC and LDL. Our findings have important clinical implications because the benefits of onion on blood lipids can effectively reduce the risk of cardiovascular disease (Mattiuzzi et al., 2020; Rader & Hovingh, 2014).

Onions are rich in flavonoids and dietary fiber, which epidemiology has shown can reduce the risk of cardiovascular disease (Hamauzu et al., 2011). However, the clinical evidence for the efficacy of onion on blood lipids is not uniform. Some studies have shown that onion consumption is effective in improving blood lipid levels in both patients with hyperlipidemia and healthy subjects (Louria et al., 1985; Vidyashankar et al., 2010). Chiu et al. (Chiu et al., 2016) found that red wine onion extract showed additional lipid‐lowering effects compared with red wine after supplementing red wine onion extract at 250 ml/day for 10 weeks for hyperlipidemia patients. Lee et al. showed that with the intake of frozen onion powder, the subjects' plasma HDL level increased, levels of plasma TC, plasma LDL, and atherosclerosis index significantly decreased (Lee et al., 2008), which was similar to our results. In addition to the changes in experimental indicators, a questionnaire for participants showed that most patients with hyperlipidemia thought onion intake was good for their health (Lee et al., 2008). However, Arora's study (Arora & Arora, 1981) did not observe the benefit of onion supplementation on dyslipidemia. Similarly, Ebrahimi‐Mamaghani et al. found that eating raw red onion did not improve HDL and TG levels in patients with polycystic ovary syndrome (Ebrahimi‐Mamaghani et al., 2014). Different characteristics of the subjects (healthy subjects, hyperlipidemia subjects), different onion products (onion skin extract, raw onion, onion juice), and Inconsistency in the duration of onion supplementation may lead to inconsistent results. As the results of the subgroup analysis showed, onion appeared to be more beneficial for subjects with dyslipidemia, and onion supplementation for longer than 10 weeks may be more beneficial for improving HDL. Since eight studies used onion extract, one used red onion, and one used onion juice, subgroup analysis based on different onion products was not feasible.

Although a large number of studies have investigated the effects of onion on blood lipids, the mechanism of onion improving blood lipids is still unclear and may be related to the following aspects. On the one hand, onion can activate lecithin‐cholesterol Acyltransferase by enhancing the action of insulin, so as to promote the conversion of LDL into HDL (Ige & Akhigbe, 2013). On the other hand, onion can promote the excretion of bile acids and inhibit the absorption of cholesterol to reduce plasma cholesterol (Guan et al., 2010). In addition, the anti‐lipid effect of onion may be related to reducing lipid hydroperoxide and lipoperoxide concentrations (Campos et al., 2003; Guan et al., 2010).

Natural products are increasingly popular because they are believed to have few side effects (Teshika et al., 2019). Onions as a common food have been the subject of numerous clinical trials, only few side‐effects have been reported. Nishimura et al. (Nishimura et al., 2019) reported four participants with mild gastrointestinal symptoms. In Song et al.'s study, two patients experienced transient itching after using the onion extract gel (Song et al., 2018). These suggest that onion administration is a safe and feasible alternative therapy.

Our study had several advantages. On the one hand, we strictly limited the inclusion criteria and only randomized placebo‐controlled trials were included. On the other hand, we conducted a comprehensive evidence search without language or time restrictions, reducing bias. Like all studies, our meta‐analysis has some limitations. First, most of the included studies were small sample studies, which may affect the reliability of the treatment effect. Second, most of the included studies were conducted in Korea, which makes it difficult to apply the results to other regions. Finally, the longest duration of onion supplementation was only 12 weeks, which may affect the true effect of onion on blood lipids.

## 5 CONCLUSIONS

In summary, onion supplementation was beneficial to control dyslipidemia, including improving plasma levels of HDL, LDL, and TC, but did not reduce plasma TG level. The therapeutic benefits of onion for dyslipidemia need to be treated with caution considering that some of the results are not robust. It is necessary to investigate the effect of long‐term onion supplementation on dyslipidemia through large‐sample, high‐quality studies. In addition, onion may be more effective in subjects with dyslipidemia, and future studies should focus on patients with hyperlipidemia to better assess the effect of onion on blood lipid.

## CONFLICTS OF INTEREST

The authors declare that they do not have any conflict of interest.

## AUTHOR CONTRIBUTIONS

**Wang Huang:** Conceptualization (equal); Investigation (equal); Methodology (equal); Software (equal); Writing‐original draft (equal); Writing‐review & editing (equal). **gang tang:** Conceptualization (equal); Data curation (equal); Formal analysis (equal); Methodology (equal); Software (equal); Writing‐original draft (equal); Writing‐review & editing (equal). **linyu zhang:** Formal analysis (equal); Methodology (equal); Software (equal); Writing‐original draft (equal); Writing‐review & editing (equal). **jie tao:** Data curation (equal); Formal analysis (equal); Methodology (equal); Software (equal); Writing‐original draft (equal); Writing‐review & editing (equal). **zhengqiang wei:** Conceptualization (equal); Formal analysis (equal); Methodology (equal); Software (equal); Writing‐review & editing (lead).

## ETHICAL APPROVAL

This study does not involve any human or animal testing.

## Data Availability

Data sharing is not applicable to this article as no new data were created or analyzed in this study.
